# [1,4-Phenyl­enebis(methyl­ene)]bis­(tri­phenyl­phospho­nium) bis­(tetra­fluoro­borate)

**DOI:** 10.1107/S1600536811048975

**Published:** 2011-11-23

**Authors:** Hamisu Ibrahim, Neil Koorbanally, Deresh Ramjugernath, Muhammad D. Bala, Vincent O. Nyamori

**Affiliations:** a School of Chemistry, University of KwaZulu-Natal, Westville Campus, Private Bag X54001, Durban 4000, South Africa; b School of Chemical Engineering, University of KwaZulu-Natal, Private Bag X54001 , Durban 4000, South Africa

## Abstract

The crystal structure of the title salt, C_44_H_38_P_2_^2+^2BF_4_^−^, consists of discrete dications inter­laced with the BF_4_^−^ counter-ions. In each cation, both phospho­nium groups lie on the same side of the plane of the central benzene ring. The tetra­fluoro­borate anions are involved in intensive thermal motion, thus some B—F bond lengths [range 1.329 (6) to 1.391 (6) Å] deviate significantly from their standard values.

## Related literature

For a related synthetic strategy, see: Ganesan & Alias (2008 ▶). For salts containing the triphenyl­phospho­nium cation, see: Kariuki *et al.* (2009 ▶). For applications of phospho­nium salts as ionic liquids, see: Cieniecka-Roslonkiewicz *et al.* (2005 ▶). For a similar mono-phospho­nium compound, see: Hafiz (2008 ▶).
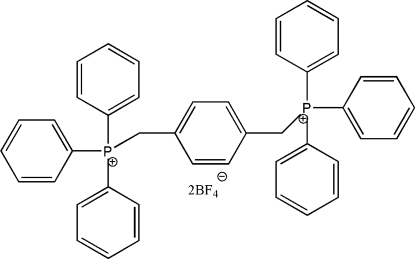


## Experimental

### 

#### Crystal data

C_44_H_38_P_2_^2+^·2BF_4_^−^
*M* *_r_* = 802.30
Monoclinic, 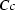

*a* = 21.8874 (5) Å
*b* = 14.4610 (3) Å
*c* = 15.2818 (3) Å
β = 123.898 (1)°
*V* = 4014.78 (15) Å^3^
*Z* = 4
Mo *K*α radiation
μ = 0.18 mm^−1^
*T* = 173 K
0.52 × 0.22 × 0.13 mm

#### Data collection

Bruker APEXII CCD area-detector diffractometer
25162 measured reflections
4853 independent reflections
3786 reflections with *I* > 2σ(*I*)
*R*_int_ = 0.058

#### Refinement

*R*[*F* ^2^ > 2σ(*F* ^2^)] = 0.046
*wR*(*F* ^2^) = 0.113
*S* = 1.10
4853 reflections
506 parameters
2 restraints
H-atom parameters constrained
Δρ_max_ = 0.43 e Å^−3^
Δρ_min_ = −0.33 e Å^−3^

### 

Data collection: *APEX2* (Bruker, 2009 ▶); cell refinement: *SAINT-Plus* (Bruker, 2009 ▶); data reduction: *SAINT-Plus*; program(s) used to solve structure: *SHELXS97* (Sheldrick, 2008 ▶); program(s) used to refine structure: *SHELXL97* (Sheldrick, 2008 ▶); molecular graphics: *ORTEP-3* (Farrugia, 1997 ▶); software used to prepare material for publication: *WinGX* (Farrugia, 1999 ▶).

## Supplementary Material

Crystal structure: contains datablock(s) global, I. DOI: 10.1107/S1600536811048975/yk2028sup1.cif

Structure factors: contains datablock(s) I. DOI: 10.1107/S1600536811048975/yk2028Isup2.hkl

Supplementary material file. DOI: 10.1107/S1600536811048975/yk2028Isup3.cml

Additional supplementary materials:  crystallographic information; 3D view; checkCIF report

## Figures and Tables

**Fig. 1.**
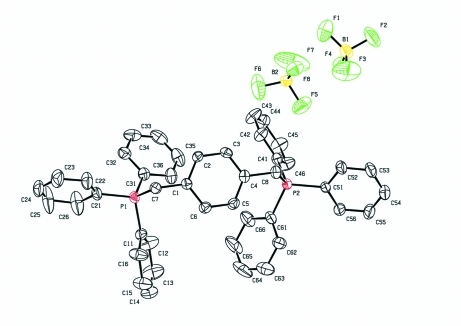
The structure of the title compound showing the atom numbering scheme and the displacement ellipsoids drawn at the 50% probability level. H atoms are omitted for clarity.

## supplementary crystallographic information

### Comment

The title compound was obtained in our effort to develop fluorinated anti microbial compounds. Preliminary investigation of related compounds, like linear phosphonium ionic liquids, has indicated activities against some strains of bacteria and fungi (*Candida albicans*) as reported by Cieniecka-Roslonkiewicz *et al.* (2005).

The compound (I) crystallizes as white block crystals in the monoclinic space group *Cc* with one dication and two BF_4_^-^ anions in the asymmetric unit. Bond lengths for C(7) – P(1) and C(8) – P(2) linking the methylene carbon atoms with the phosphorus atoms are 1.811 (3) and 1.817 (3) Å respectively, which is comparable with the 1.817 (2) Å reported by Hafiz (2008) for a related mono-phosphonium compound.

### Experimental

The compound was synthesized according to the slightly modified method reported by Ganesan & Alias (2008). α,α-Dibromo-*p*-xylene (0.528 g, 0.002 moles) was dissolved in distilled anhydrous dichloromethane. Triphenylphosphine (1.049 g, 0.004 moles) was added to this solution, and the mixture was stirred for 24 hrs at room temperature to afford the quaternized salt. The solvent was removed *in vacuo*, the residue washed with acetone to remove unreacted material. The washed residue was filtered and dried, yielding white precipitate of the dicationic phosphonium salt. The salt was then dissolved in a mixture of water and ethanol (1:4) and the salt NaBF_4_ was added (1:2). The mixture was stirred for 4 hrs at room temperature, dried with MgSO_4_ and concentrated. The solid residue was extracted with ethanol, and the solvent was evaporated. The yield of (I) was 79.8%. X-ray quality crystals were grown from ethanol solution, m.p. 295 °C (decomp).

^1^H-NMR (600 MHz, DMSO-d6, 323 K), (δ_H_, p.p.m.): 5.08 (d, *J* = 14.69 Hz, 4H, Ar—CH_2_), 6.76 (s, 4H, Ar—H), 7.68 (m, 24H, Ar—H), and 7.90 (m, 6H, Ar—H).

^13^C-NMR (400 MHz, DMSO-d6, 323 K), (δ_c_, p.p.m.): 27.66 (d, *J* = 50.26 Hz, (methylene)), 117.51 (d, *J* =85.90 Hz, C-1'), 128.12 (d, *J* = 4.04 Hz, C-1/4), 130.02 (d, *J* = 6.29 Hz, C-3'/5')^*^, 130.09 (d, *J* = 6.29 Hz, C-3'/5')^*^, 131.11 (s, C-2/3/5/6/), 133.86 (d, *J* = 4.89 Hz, C-2'/6')^*^, 133.91 (d, *J* = 4.89 Hz, C-2'/6')^*^, 135.13 (s, C-4'). ^*^The two triphenylphosphine groups are not exactly equivalent and hence two sets of resonances are seen for C-2'/6') and C-3'/5').

^19^F NMR (400 MHz, DMSO-d6, 298 K), (δ_F_, p.p.m.): -148.22 (^10^BF_4_^-^), -148.27 (^11^BF_4_^-^).

^31^P NMR (162 MHz, DMSO-D_6_, 298 K), (δ_p_, p.p.m.): 23.07.

FT—IR (cm^-1^): 3062, 2977, 2935, 1438, 1111, 1046.

### Refinement

All H-atoms were refined using a riding model, with C—H = 0.95 Å and *U*_iso_(H) = 1.2*U*_eq_(C) for aromatic, C—H = 0.99 Å and *U*_iso_(H) = 1.2*U*_eq_(C) for CH_2_.

In the absence of significant anomalous scattering effects Friedel pairs have been merged.

### Crystal data

**Table:**

C_44_H_38_P_2_^2+^·2BF_4_^−^	*F*(000) = 1656
*M**_r_* = 802.30	*D*_x_ = 1.327 Mg m^−^^3^
Monoclinic, *C**c*	Mo *K*α radiation, λ = 0.71073 Å
Hall symbol: C -2yc	Cell parameters from 5943 reflections
*a* = 21.8874 (5) Å	θ = 2.7–24.0°
*b* = 14.4610 (3) Å	µ = 0.18 mm^−^^1^
*c* = 15.2818 (3) Å	*T* = 173 K
β = 123.898 (1)°	Needle, colourless
*V* = 4014.78 (15) Å^3^	0.52 × 0.22 × 0.13 mm
*Z* = 4	

### Data collection

**Table:**

Bruker APEXII CCD area-detector diffractometer	3786 reflections with *I* > 2σ(*I*)
Radiation source: fine-focus sealed tube	*R*_int_ = 0.058
graphite	θ_max_ = 28.0°, θ_min_ = 1.8°
φ and ω scans	*h* = −28→28
25162 measured reflections	*k* = −19→18
4853 independent reflections	*l* = −20→20

### Refinement

**Table:**

Refinement on *F*^2^	Primary atom site location: structure-invariant direct methods
Least-squares matrix: full	Secondary atom site location: difference Fourier map
*R*[*F*^2^ > 2σ(*F*^2^)] = 0.046	Hydrogen site location: inferred from neighbouring sites
*wR*(*F*^2^) = 0.113	H-atom parameters constrained
*S* = 1.10	*w* = 1/[σ^2^(*F*_o_^2^) + (0.0571*P*)^2^] where *P* = (*F*_o_^2^ + 2*F*_c_^2^)/3
4853 reflections	(Δ/σ)_max_ = 0.032
506 parameters	Δρ_max_ = 0.43 e Å^−^^3^
2 restraints	Δρ_min_ = −0.33 e Å^−^^3^

### Special details

**Table:**

Geometry. All s.u.'s (except the s.u. in the dihedral angle between two l.s. planes) are estimated using the full covariance matrix. The cell s.u.'s are taken into account individually in the estimation of s.u.'s in distances, angles and torsion angles; correlations between s.u.'s in cell parameters are only used when they are defined by crystal symmetry. An approximate (isotropic) treatment of cell s.u.'s is used for estimating s.u.'s involving l.s. planes.
Refinement. Refinement of *F*^2^ against ALL reflections. The weighted *R*-factor *wR* and goodness of fit *S* are based on *F*^2^, conventional *R*-factors *R* are based on *F*, with *F* set to zero for negative *F*^2^. The threshold expression of *F*^2^ > σ(*F*^2^) is used only for calculating *R*-factors(gt) *etc*. and is not relevant to the choice of reflections for refinement. *R*-factors based on *F*^2^ are statistically about twice as large as those based on *F*, and *R*- factors based on ALL data will be even larger.

### Fractional atomic coordinates and isotropic or equivalent isotropic displacement parameters (Å^2^)

**Table:**

	*x*	*y*	*z*	*U*_iso_*/*U*_eq_	
C1	0.29567 (19)	0.8761 (2)	0.3995 (3)	0.0254 (7)	
C2	0.35338 (19)	0.8962 (2)	0.3896 (3)	0.0278 (7)	
H2	0.4001	0.9118	0.4507	0.033*	
C3	0.34422 (19)	0.8942 (2)	0.2920 (3)	0.0290 (7)	
H3	0.3845	0.9085	0.2868	0.035*	
C4	0.2764 (2)	0.8712 (2)	0.2019 (3)	0.0271 (8)	
C5	0.2178 (2)	0.8531 (3)	0.2112 (3)	0.0328 (8)	
H5	0.1707	0.8393	0.1498	0.039*	
C6	0.22707 (19)	0.8549 (3)	0.3082 (3)	0.0314 (8)	
H6	0.1866	0.8416	0.3131	0.038*	
C7	0.3067 (2)	0.8733 (2)	0.5052 (3)	0.0291 (8)	
H7A	0.2626	0.8990	0.4992	0.035*	
H7B	0.3493	0.9128	0.5548	0.035*	
C8	0.2650 (2)	0.8716 (2)	0.0939 (3)	0.0303 (8)	
H8A	0.3084	0.9013	0.1014	0.036*	
H8B	0.2218	0.9110	0.0463	0.036*	
C11	0.2368 (2)	0.6952 (2)	0.4900 (3)	0.0318 (8)	
C12	0.2281 (2)	0.6090 (3)	0.4461 (4)	0.0579 (13)	
H12	0.2684	0.5795	0.4500	0.069*	
C13	0.1602 (3)	0.5654 (3)	0.3962 (4)	0.0693 (16)	
H13	0.1535	0.5072	0.3632	0.083*	
C14	0.1027 (3)	0.6053 (3)	0.3939 (4)	0.0546 (12)	
H14	0.0574	0.5732	0.3638	0.066*	
C15	0.1109 (3)	0.6928 (3)	0.4358 (4)	0.0609 (13)	
H15	0.0705	0.7222	0.4314	0.073*	
C16	0.1782 (2)	0.7373 (3)	0.4840 (4)	0.0545 (12)	
H16	0.1840	0.7972	0.5132	0.065*	
C21	0.3574 (2)	0.7610 (3)	0.6955 (3)	0.0334 (9)	
C22	0.3793 (3)	0.8412 (3)	0.7521 (3)	0.0498 (11)	
H22	0.3768	0.8978	0.7187	0.060*	
C23	0.4056 (3)	0.8405 (4)	0.8588 (3)	0.0586 (13)	
H23	0.4201	0.8967	0.8975	0.070*	
C24	0.4103 (3)	0.7607 (4)	0.9069 (3)	0.0543 (12)	
H24	0.4260	0.7608	0.9788	0.065*	
C25	0.3925 (4)	0.6795 (4)	0.8526 (4)	0.093 (2)	
H25	0.3998	0.6229	0.8888	0.112*	
C26	0.3641 (4)	0.6779 (4)	0.7460 (4)	0.0770 (18)	
H26	0.3494	0.6213	0.7080	0.092*	
C31	0.3883 (2)	0.6991 (2)	0.5404 (3)	0.0309 (8)	
C32	0.4589 (2)	0.6802 (3)	0.6277 (3)	0.0391 (9)	
H32	0.4727	0.6970	0.6965	0.047*	
C33	0.5089 (2)	0.6361 (3)	0.6123 (4)	0.0492 (11)	
H33	0.5568	0.6215	0.6714	0.059*	
C34	0.4900 (3)	0.6137 (3)	0.5133 (4)	0.0516 (12)	
H34	0.5248	0.5844	0.5040	0.062*	
C35	0.4212 (3)	0.6332 (3)	0.4280 (4)	0.0510 (11)	
H35	0.4086	0.6173	0.3595	0.061*	
C36	0.3697 (2)	0.6759 (3)	0.4396 (3)	0.0400 (9)	
H36	0.3220	0.6894	0.3795	0.048*	
C41	0.3354 (2)	0.6988 (2)	0.0830 (3)	0.0287 (8)	
C42	0.4020 (2)	0.7307 (3)	0.1686 (3)	0.0385 (9)	
H42	0.4043	0.7878	0.2011	0.046*	
C43	0.4653 (2)	0.6789 (3)	0.2064 (3)	0.0521 (12)	
H43	0.5108	0.7006	0.2655	0.062*	
C44	0.4634 (2)	0.5968 (3)	0.1599 (3)	0.0438 (10)	
H44	0.5071	0.5617	0.1872	0.053*	
C45	0.3975 (2)	0.5654 (3)	0.0733 (3)	0.0483 (11)	
H45	0.3958	0.5089	0.0402	0.058*	
C46	0.3340 (2)	0.6168 (3)	0.0349 (3)	0.0452 (10)	
H46	0.2889	0.5956	−0.0253	0.054*	
C51	0.21135 (19)	0.7844 (2)	−0.1065 (3)	0.0257 (7)	
C52	0.2345 (2)	0.8608 (3)	−0.1341 (3)	0.0384 (9)	
H52	0.2723	0.8992	−0.0809	0.046*	
C53	0.2026 (2)	0.8817 (3)	−0.2394 (3)	0.0453 (10)	
H53	0.2182	0.9349	−0.2584	0.054*	
C54	0.1484 (2)	0.8259 (3)	−0.3166 (3)	0.0447 (10)	
H54	0.1257	0.8417	−0.3887	0.054*	
C55	0.1271 (2)	0.7475 (3)	−0.2899 (3)	0.0482 (11)	
H55	0.0912	0.7077	−0.3436	0.058*	
C56	0.1577 (2)	0.7263 (3)	−0.1851 (3)	0.0368 (8)	
H56	0.1425	0.6724	−0.1666	0.044*	
C61	0.1912 (2)	0.6890 (3)	0.0463 (3)	0.0327 (8)	
C62	0.1165 (2)	0.7106 (3)	−0.0065 (3)	0.0401 (9)	
H62	0.0967	0.7608	−0.0548	0.048*	
C63	0.0715 (3)	0.6593 (4)	0.0112 (4)	0.0577 (13)	
H63	0.0205	0.6734	−0.0256	0.069*	
C64	0.1005 (3)	0.5871 (4)	0.0827 (5)	0.0771 (18)	
H64	0.0693	0.5519	0.0950	0.093*	
C65	0.1740 (3)	0.5657 (4)	0.1361 (5)	0.0755 (16)	
H65	0.1934	0.5161	0.1852	0.091*	
C66	0.2201 (3)	0.6165 (3)	0.1186 (4)	0.0533 (11)	
H66	0.2710	0.6019	0.1558	0.064*	
B1	0.1000 (3)	0.9769 (3)	0.4235 (4)	0.0422 (11)	
B2	0.4719 (2)	1.0386 (3)	0.6578 (3)	0.0331 (10)	
F1	0.16257 (17)	0.95422 (19)	0.5214 (2)	0.0692 (8)	
F2	0.1130 (2)	0.9491 (3)	0.3495 (3)	0.0983 (12)	
F3	0.0411 (2)	0.9335 (3)	0.4089 (4)	0.1183 (16)	
F4	0.09095 (14)	1.07117 (18)	0.4166 (2)	0.0594 (7)	
F5	0.40535 (15)	1.0708 (2)	0.5748 (2)	0.0770 (10)	
F6	0.4802 (3)	1.0623 (3)	0.7481 (3)	0.144 (2)	
F7	0.5246 (3)	1.0771 (3)	0.6519 (5)	0.143 (2)	
F8	0.47676 (13)	0.94392 (17)	0.6507 (2)	0.0544 (7)	
P1	0.32289 (5)	0.75698 (6)	0.55775 (6)	0.0273 (2)	
P2	0.25107 (5)	0.76029 (6)	0.03033 (6)	0.0247 (2)	

### Atomic displacement parameters (Å^2^)

**Table:**

	*U*^11^	*U*^22^	*U*^33^	*U*^12^	*U*^13^	*U*^23^
C1	0.0266 (18)	0.0248 (17)	0.0266 (18)	0.0027 (14)	0.0160 (16)	0.0003 (13)
C2	0.0271 (17)	0.0296 (17)	0.0226 (17)	−0.0056 (14)	0.0113 (14)	−0.0037 (14)
C3	0.0296 (18)	0.0264 (17)	0.036 (2)	−0.0058 (14)	0.0215 (16)	−0.0030 (14)
C4	0.033 (2)	0.0223 (17)	0.0261 (18)	0.0062 (14)	0.0167 (16)	0.0025 (13)
C5	0.0236 (17)	0.045 (2)	0.0272 (18)	0.0038 (16)	0.0126 (15)	−0.0030 (16)
C6	0.0271 (18)	0.036 (2)	0.0316 (19)	0.0000 (15)	0.0166 (16)	−0.0050 (16)
C7	0.0310 (19)	0.0296 (19)	0.0286 (19)	−0.0041 (15)	0.0178 (16)	−0.0046 (14)
C8	0.037 (2)	0.0280 (19)	0.0283 (19)	0.0034 (15)	0.0193 (17)	0.0001 (15)
C11	0.033 (2)	0.0305 (19)	0.0281 (19)	−0.0064 (16)	0.0143 (17)	−0.0025 (15)
C12	0.038 (2)	0.038 (2)	0.080 (3)	−0.0002 (19)	0.022 (2)	−0.016 (2)
C13	0.057 (3)	0.044 (3)	0.090 (4)	−0.015 (2)	0.031 (3)	−0.029 (3)
C14	0.041 (2)	0.056 (3)	0.056 (3)	−0.020 (2)	0.021 (2)	−0.012 (2)
C15	0.048 (3)	0.064 (3)	0.081 (4)	−0.017 (2)	0.042 (3)	−0.015 (3)
C16	0.051 (3)	0.053 (3)	0.078 (3)	−0.018 (2)	0.048 (3)	−0.029 (2)
C21	0.041 (2)	0.039 (2)	0.0264 (19)	−0.0021 (17)	0.0219 (17)	−0.0015 (15)
C22	0.072 (3)	0.047 (2)	0.031 (2)	−0.007 (2)	0.029 (2)	−0.0030 (18)
C23	0.074 (3)	0.066 (3)	0.034 (2)	−0.010 (3)	0.029 (2)	−0.013 (2)
C24	0.054 (3)	0.085 (4)	0.025 (2)	0.006 (2)	0.023 (2)	0.003 (2)
C25	0.171 (7)	0.071 (4)	0.054 (3)	0.014 (4)	0.073 (4)	0.020 (3)
C26	0.142 (5)	0.048 (3)	0.054 (3)	−0.007 (3)	0.062 (4)	0.002 (2)
C31	0.036 (2)	0.0264 (18)	0.032 (2)	0.0013 (15)	0.0197 (17)	0.0016 (15)
C32	0.037 (2)	0.046 (2)	0.040 (2)	−0.0042 (18)	0.0249 (19)	−0.0035 (18)
C33	0.033 (2)	0.048 (3)	0.069 (3)	0.0001 (18)	0.030 (2)	0.003 (2)
C34	0.061 (3)	0.044 (2)	0.078 (3)	0.003 (2)	0.056 (3)	0.002 (2)
C35	0.083 (3)	0.039 (2)	0.055 (3)	0.006 (2)	0.053 (3)	0.002 (2)
C36	0.055 (2)	0.0337 (19)	0.035 (2)	0.0070 (18)	0.0278 (19)	0.0019 (15)
C41	0.033 (2)	0.0256 (17)	0.0287 (19)	0.0011 (15)	0.0183 (17)	0.0024 (14)
C42	0.033 (2)	0.038 (2)	0.034 (2)	0.0044 (16)	0.0113 (17)	−0.0027 (16)
C43	0.031 (2)	0.061 (3)	0.039 (2)	0.0088 (19)	0.0042 (19)	−0.006 (2)
C44	0.033 (2)	0.046 (2)	0.047 (2)	0.0119 (19)	0.0190 (19)	0.0058 (19)
C45	0.042 (2)	0.036 (2)	0.057 (3)	0.0065 (18)	0.022 (2)	−0.0081 (19)
C46	0.036 (2)	0.038 (2)	0.053 (3)	−0.0011 (17)	0.0190 (19)	−0.0121 (18)
C51	0.0268 (18)	0.0290 (17)	0.0237 (17)	0.0011 (14)	0.0156 (15)	0.0022 (14)
C52	0.050 (2)	0.041 (2)	0.0285 (19)	−0.0148 (18)	0.0248 (19)	−0.0074 (16)
C53	0.062 (3)	0.045 (2)	0.037 (2)	−0.009 (2)	0.033 (2)	0.0044 (17)
C54	0.047 (2)	0.059 (3)	0.029 (2)	0.001 (2)	0.0222 (19)	0.0041 (19)
C55	0.053 (3)	0.053 (3)	0.026 (2)	−0.014 (2)	0.0148 (19)	−0.0028 (17)
C56	0.040 (2)	0.038 (2)	0.0288 (19)	−0.0120 (17)	0.0168 (17)	−0.0035 (15)
C61	0.043 (2)	0.032 (2)	0.032 (2)	−0.0051 (17)	0.0267 (18)	−0.0015 (16)
C62	0.038 (2)	0.043 (2)	0.044 (2)	−0.0062 (18)	0.0258 (19)	−0.0022 (18)
C63	0.054 (3)	0.063 (3)	0.073 (3)	−0.019 (2)	0.045 (3)	−0.019 (3)
C64	0.093 (4)	0.074 (4)	0.106 (5)	−0.028 (3)	0.081 (4)	−0.002 (3)
C65	0.091 (4)	0.073 (4)	0.091 (4)	−0.001 (3)	0.069 (4)	0.030 (3)
C66	0.064 (3)	0.050 (3)	0.061 (3)	0.004 (2)	0.044 (2)	0.017 (2)
B1	0.045 (3)	0.045 (3)	0.045 (3)	−0.007 (2)	0.030 (2)	−0.012 (2)
B2	0.027 (2)	0.037 (2)	0.034 (2)	0.0011 (18)	0.0156 (19)	−0.0049 (18)
F1	0.089 (2)	0.0490 (15)	0.0475 (15)	−0.0135 (15)	0.0243 (15)	0.0046 (12)
F2	0.134 (3)	0.115 (3)	0.078 (2)	0.057 (2)	0.079 (2)	0.0109 (19)
F3	0.066 (2)	0.099 (3)	0.182 (4)	−0.042 (2)	0.064 (3)	−0.028 (3)
F4	0.0533 (16)	0.0487 (15)	0.0649 (18)	0.0071 (12)	0.0260 (14)	−0.0031 (13)
F5	0.0611 (18)	0.0594 (18)	0.065 (2)	0.0157 (15)	0.0068 (15)	−0.0042 (14)
F6	0.225 (5)	0.139 (3)	0.054 (2)	0.128 (4)	0.070 (3)	0.030 (2)
F7	0.121 (3)	0.063 (2)	0.311 (7)	−0.022 (2)	0.162 (4)	−0.015 (3)
F8	0.0449 (14)	0.0435 (13)	0.0596 (16)	0.0008 (11)	0.0197 (12)	−0.0028 (12)
P1	0.0313 (5)	0.0290 (5)	0.0239 (5)	−0.0035 (4)	0.0168 (4)	−0.0032 (4)
P2	0.0248 (4)	0.0276 (4)	0.0226 (4)	0.0006 (4)	0.0137 (4)	0.0012 (4)

### Geometric parameters (Å, °)

**Table:**

C1—C2	1.384 (5)	C34—C35	1.362 (7)
C1—C6	1.399 (5)	C34—H34	0.9500
C1—C7	1.495 (5)	C35—C36	1.381 (6)
C2—C3	1.391 (5)	C35—H35	0.9500
C2—H2	0.9500	C36—H36	0.9500
C3—C4	1.390 (5)	C41—C42	1.386 (5)
C3—H3	0.9500	C41—C46	1.387 (5)
C4—C5	1.392 (5)	C41—P2	1.785 (4)
C4—C8	1.525 (5)	C42—C43	1.387 (5)
C5—C6	1.380 (5)	C42—H42	0.9500
C5—H5	0.9500	C43—C44	1.372 (6)
C6—H6	0.9500	C43—H43	0.9500
C7—P1	1.812 (4)	C44—C45	1.381 (6)
C7—H7A	0.9900	C44—H44	0.9500
C7—H7B	0.9900	C45—C46	1.386 (6)
C8—P2	1.817 (4)	C45—H45	0.9500
C8—H8A	0.9900	C46—H46	0.9500
C8—H8B	0.9900	C51—C52	1.377 (5)
C11—C16	1.376 (6)	C51—C56	1.398 (5)
C11—C12	1.378 (6)	C51—P2	1.795 (3)
C11—P1	1.800 (4)	C52—C53	1.384 (5)
C12—C13	1.387 (6)	C52—H52	0.9500
C12—H12	0.9500	C53—C54	1.374 (6)
C13—C14	1.367 (7)	C53—H53	0.9500
C13—H13	0.9500	C54—C55	1.371 (6)
C14—C15	1.382 (7)	C54—H54	0.9500
C14—H14	0.9500	C55—C56	1.383 (5)
C15—C16	1.385 (6)	C55—H55	0.9500
C15—H15	0.9500	C56—H56	0.9500
C16—H16	0.9500	C61—C66	1.393 (6)
C21—C22	1.363 (6)	C61—C62	1.398 (5)
C21—C26	1.391 (6)	C61—P2	1.788 (4)
C21—P1	1.797 (4)	C62—C63	1.376 (6)
C22—C23	1.395 (6)	C62—H62	0.9500
C22—H22	0.9500	C63—C64	1.383 (8)
C23—C24	1.340 (7)	C63—H63	0.9500
C23—H23	0.9500	C64—C65	1.372 (8)
C24—C25	1.364 (8)	C64—H64	0.9500
C24—H24	0.9500	C65—C66	1.389 (6)
C25—C26	1.383 (7)	C65—H65	0.9500
C25—H25	0.9500	C66—H66	0.9500
C26—H26	0.9500	B1—F3	1.336 (6)
C31—C32	1.393 (5)	B1—F2	1.371 (6)
C31—C36	1.398 (5)	B1—F4	1.374 (6)
C31—P1	1.801 (4)	B1—F1	1.391 (6)
C32—C33	1.397 (6)	B2—F7	1.329 (6)
C32—H32	0.9500	B2—F6	1.331 (6)
C33—C34	1.365 (7)	B2—F5	1.373 (5)
C33—H33	0.9500	B2—F8	1.383 (5)
			
C2—C1—C6	118.3 (3)	C35—C36—C31	119.5 (4)
C2—C1—C7	121.0 (3)	C35—C36—H36	120.2
C6—C1—C7	120.6 (3)	C31—C36—H36	120.2
C1—C2—C3	121.2 (3)	C42—C41—C46	119.0 (3)
C1—C2—H2	119.4	C42—C41—P2	122.6 (3)
C3—C2—H2	119.4	C46—C41—P2	118.4 (3)
C2—C3—C4	120.2 (3)	C41—C42—C43	119.6 (4)
C2—C3—H3	119.9	C41—C42—H42	120.2
C4—C3—H3	119.9	C43—C42—H42	120.2
C5—C4—C3	118.7 (3)	C44—C43—C42	121.1 (4)
C5—C4—C8	120.5 (3)	C44—C43—H43	119.5
C3—C4—C8	120.7 (3)	C42—C43—H43	119.5
C6—C5—C4	120.9 (3)	C43—C44—C45	119.7 (4)
C6—C5—H5	119.5	C43—C44—H44	120.1
C4—C5—H5	119.5	C45—C44—H44	120.1
C5—C6—C1	120.6 (3)	C44—C45—C46	119.5 (4)
C5—C6—H6	119.7	C44—C45—H45	120.2
C1—C6—H6	119.7	C46—C45—H45	120.2
C1—C7—P1	112.2 (2)	C45—C46—C41	121.0 (4)
C1—C7—H7A	109.2	C45—C46—H46	119.5
P1—C7—H7A	109.2	C41—C46—H46	119.5
C1—C7—H7B	109.2	C52—C51—C56	119.7 (3)
P1—C7—H7B	109.2	C52—C51—P2	119.4 (3)
H7A—C7—H7B	107.9	C56—C51—P2	120.9 (3)
C4—C8—P2	116.9 (2)	C51—C52—C53	119.9 (4)
C4—C8—H8A	108.1	C51—C52—H52	120.1
P2—C8—H8A	108.1	C53—C52—H52	120.1
C4—C8—H8B	108.1	C54—C53—C52	120.3 (4)
P2—C8—H8B	108.1	C54—C53—H53	119.9
H8A—C8—H8B	107.3	C52—C53—H53	119.9
C16—C11—C12	119.8 (4)	C55—C54—C53	120.3 (4)
C16—C11—P1	117.1 (3)	C55—C54—H54	119.9
C12—C11—P1	123.0 (3)	C53—C54—H54	119.9
C11—C12—C13	119.5 (4)	C54—C55—C56	120.2 (4)
C11—C12—H12	120.2	C54—C55—H55	119.9
C13—C12—H12	120.2	C56—C55—H55	119.9
C14—C13—C12	120.8 (4)	C55—C56—C51	119.6 (3)
C14—C13—H13	119.6	C55—C56—H56	120.2
C12—C13—H13	119.6	C51—C56—H56	120.2
C13—C14—C15	119.5 (4)	C66—C61—C62	119.6 (4)
C13—C14—H14	120.2	C66—C61—P2	120.0 (3)
C15—C14—H14	120.2	C62—C61—P2	120.2 (3)
C16—C15—C14	119.9 (5)	C63—C62—C61	120.1 (4)
C16—C15—H15	120.1	C63—C62—H62	120.0
C14—C15—H15	120.1	C61—C62—H62	120.0
C11—C16—C15	120.3 (4)	C62—C63—C64	120.0 (5)
C11—C16—H16	119.8	C62—C63—H63	120.0
C15—C16—H16	119.8	C64—C63—H63	120.0
C22—C21—C26	119.4 (4)	C65—C64—C63	120.6 (5)
C22—C21—P1	122.7 (3)	C65—C64—H64	119.7
C26—C21—P1	117.8 (3)	C63—C64—H64	119.7
C21—C22—C23	120.4 (4)	C64—C65—C66	120.2 (5)
C21—C22—H22	119.8	C64—C65—H65	119.9
C23—C22—H22	119.8	C66—C65—H65	119.9
C24—C23—C22	120.2 (5)	C65—C66—C61	119.6 (5)
C24—C23—H23	119.9	C65—C66—H66	120.2
C22—C23—H23	119.9	C61—C66—H66	120.2
C23—C24—C25	119.9 (4)	F3—B1—F2	110.4 (4)
C23—C24—H24	120.0	F3—B1—F4	111.6 (4)
C25—C24—H24	120.0	F2—B1—F4	108.3 (4)
C24—C25—C26	121.3 (5)	F3—B1—F1	110.4 (5)
C24—C25—H25	119.4	F2—B1—F1	106.6 (4)
C26—C25—H25	119.4	F4—B1—F1	109.3 (4)
C25—C26—C21	118.6 (5)	F7—B2—F6	109.9 (5)
C25—C26—H26	120.7	F7—B2—F5	108.0 (4)
C21—C26—H26	120.7	F6—B2—F5	109.4 (4)
C32—C31—C36	119.6 (3)	F7—B2—F8	107.5 (4)
C32—C31—P1	119.9 (3)	F6—B2—F8	111.4 (4)
C36—C31—P1	120.5 (3)	F5—B2—F8	110.6 (3)
C33—C32—C31	118.9 (4)	C21—P1—C11	108.16 (18)
C33—C32—H32	120.5	C21—P1—C31	109.73 (18)
C31—C32—H32	120.5	C11—P1—C31	110.33 (18)
C34—C33—C32	120.9 (4)	C21—P1—C7	109.83 (18)
C34—C33—H33	119.6	C11—P1—C7	108.83 (18)
C32—C33—H33	119.6	C31—P1—C7	109.92 (17)
C35—C34—C33	120.1 (4)	C41—P2—C61	107.66 (17)
C35—C34—H34	119.9	C41—P2—C51	109.23 (16)
C33—C34—H34	119.9	C61—P2—C51	110.82 (17)
C34—C35—C36	121.0 (4)	C41—P2—C8	112.16 (17)
C34—C35—H35	119.5	C61—P2—C8	110.80 (18)
C36—C35—H35	119.5	C51—P2—C8	106.19 (16)
			
C6—C1—C2—C3	−1.0 (5)	C52—C51—C56—C55	−1.7 (6)
C7—C1—C2—C3	176.6 (3)	P2—C51—C56—C55	178.8 (3)
C1—C2—C3—C4	−0.3 (5)	C66—C61—C62—C63	−1.4 (6)
C2—C3—C4—C5	1.8 (5)	P2—C61—C62—C63	−175.7 (3)
C2—C3—C4—C8	178.1 (3)	C61—C62—C63—C64	1.0 (7)
C3—C4—C5—C6	−2.0 (5)	C62—C63—C64—C65	−0.2 (8)
C8—C4—C5—C6	−178.3 (3)	C63—C64—C65—C66	−0.2 (9)
C4—C5—C6—C1	0.7 (6)	C64—C65—C66—C61	−0.2 (8)
C2—C1—C6—C5	0.8 (5)	C62—C61—C66—C65	1.0 (7)
C7—C1—C6—C5	−176.9 (3)	P2—C61—C66—C65	175.3 (4)
C2—C1—C7—P1	−96.2 (4)	C22—C21—P1—C11	−129.0 (4)
C6—C1—C7—P1	81.5 (4)	C26—C21—P1—C11	53.3 (5)
C5—C4—C8—P2	−72.5 (4)	C22—C21—P1—C31	110.6 (4)
C3—C4—C8—P2	111.3 (3)	C26—C21—P1—C31	−67.1 (4)
C16—C11—C12—C13	0.0 (7)	C22—C21—P1—C7	−10.3 (4)
P1—C11—C12—C13	179.2 (4)	C26—C21—P1—C7	171.9 (4)
C11—C12—C13—C14	−2.8 (8)	C16—C11—P1—C21	68.4 (4)
C12—C13—C14—C15	4.3 (8)	C12—C11—P1—C21	−110.8 (4)
C13—C14—C15—C16	−3.1 (8)	C16—C11—P1—C31	−171.6 (3)
C12—C11—C16—C15	1.1 (7)	C12—C11—P1—C31	9.2 (4)
P1—C11—C16—C15	−178.1 (4)	C16—C11—P1—C7	−50.9 (4)
C14—C15—C16—C11	0.4 (8)	C12—C11—P1—C7	129.9 (4)
C26—C21—C22—C23	−2.5 (7)	C32—C31—P1—C21	−11.2 (4)
P1—C21—C22—C23	179.8 (4)	C36—C31—P1—C21	171.1 (3)
C21—C22—C23—C24	0.9 (8)	C32—C31—P1—C11	−130.3 (3)
C22—C23—C24—C25	2.9 (8)	C36—C31—P1—C11	52.0 (3)
C23—C24—C25—C26	−5.1 (10)	C32—C31—P1—C7	109.7 (3)
C24—C25—C26—C21	3.4 (10)	C36—C31—P1—C7	−68.0 (3)
C22—C21—C26—C25	0.4 (9)	C1—C7—P1—C21	166.1 (3)
P1—C21—C26—C25	178.2 (5)	C1—C7—P1—C11	−75.6 (3)
C36—C31—C32—C33	−1.6 (5)	C1—C7—P1—C31	45.3 (3)
P1—C31—C32—C33	−179.3 (3)	C42—C41—P2—C61	−117.1 (3)
C31—C32—C33—C34	1.5 (6)	C46—C41—P2—C61	64.2 (3)
C32—C33—C34—C35	−0.8 (7)	C42—C41—P2—C51	122.5 (3)
C33—C34—C35—C36	0.1 (7)	C46—C41—P2—C51	−56.2 (3)
C34—C35—C36—C31	−0.2 (6)	C42—C41—P2—C8	5.1 (4)
C32—C31—C36—C35	1.0 (6)	C46—C41—P2—C8	−173.7 (3)
P1—C31—C36—C35	178.7 (3)	C66—C61—P2—C41	18.8 (4)
C46—C41—C42—C43	−2.1 (6)	C62—C61—P2—C41	−166.9 (3)
P2—C41—C42—C43	179.2 (3)	C66—C61—P2—C51	138.2 (3)
C41—C42—C43—C44	0.6 (7)	C62—C61—P2—C51	−47.5 (4)
C42—C43—C44—C45	0.7 (7)	C66—C61—P2—C8	−104.2 (4)
C43—C44—C45—C46	−0.6 (7)	C62—C61—P2—C8	70.1 (4)
C44—C45—C46—C41	−0.8 (7)	C52—C51—P2—C41	−83.8 (3)
C42—C41—C46—C45	2.2 (6)	C56—C51—P2—C41	95.7 (3)
P2—C41—C46—C45	−179.0 (4)	C52—C51—P2—C61	157.7 (3)
C56—C51—C52—C53	2.5 (6)	C56—C51—P2—C61	−22.8 (4)
P2—C51—C52—C53	−177.9 (3)	C52—C51—P2—C8	37.3 (4)
C51—C52—C53—C54	−0.7 (7)	C56—C51—P2—C8	−143.2 (3)
C52—C53—C54—C55	−2.0 (7)	C4—C8—P2—C41	−78.5 (3)
C53—C54—C55—C56	2.8 (7)	C4—C8—P2—C61	41.8 (3)
C54—C55—C56—C51	−0.9 (7)	C4—C8—P2—C51	162.2 (3)
